# The role of economic evaluation in modelling public health and social measures for pandemic policy: a systematic review

**DOI:** 10.1186/s12962-024-00585-6

**Published:** 2024-11-01

**Authors:** Shania Rossiter, Samantha Howe, Joshua Szanyi, James M. Trauer, Tim Wilson, Tony Blakely

**Affiliations:** 1 Population Interventions Unit, Centre for Epidemiology and Biostatistics, Melbourne School of Population and Global Health, The University of Melbourne, Melbourne, Australia; 2 Epidemiological Modelling Unit, School of Public Health and Preventive Medicine, Monash University, Melbourne, Australia

**Keywords:** Pandemics, Policy making, Dynamic mathematical models, Systematic review

## Abstract

**Background:**

Dynamic transmission models are often used to provide epidemiological guidance for pandemic policy decisions. However, how economic evaluation is typically incorporated into this technique to generate cost-effectiveness estimates of pandemic policy responses has not previously been reviewed.

**Methods:**

We systematically searched the Embase, PubMed and Scopus databases for dynamic epidemiological modelling studies that incorporated economic evaluation of public health and social measures (PHSMs), with no date restrictions, on 7 July 2024.

**Results:**

Of the 2,719 screened studies, 51 met the inclusion criteria. Most studies (n = 42, 82%) modelled SARS-CoV-2. A range of PHSMs were examined, including school closures, testing/screening, social distancing and mask use. Half of the studies utilised an extension of a Susceptible-Exposed-Infectious-Recovered (SEIR) compartmental model. The most common type of economic evaluation was cost-effectiveness analysis (n = 24, 47%), followed by cost-utility analysis (n = 17, 33%) and cost–benefit analysis (n = 17, 33%).

**Conclusions:**

Economic evaluation is infrequently incorporated into dynamic epidemiological modelling studies of PHSMs. The scope of this research should be expanded, given the substantial cost implications of pandemic PHSM policy responses.

**Supplementary Information:**

The online version contains supplementary material available at 10.1186/s12962-024-00585-6.

## Background

Public health and social measures (PHSMs, also referred to as non-pharmaceutical interventions) are implemented during pandemics to suppress or eliminate the transmission of infectious diseases. PHSMs are utilised when vaccines and pharmaceutical treatments are unavailable or insufficient to control the spread of the infectious agent [1]. PHSMs—particularly restrictions on social mobility and lockdowns—can yield significant benefits for population health and health system expenditure; however, they may also result in substantial social costs. Consequently, decision-making regarding the implementation and timing of PHSMs is complex. During the COVID-19 pandemic, the prevailing approach involved using simulation modelling of the health impacts of PHSMs, sometimes compared with parallel estimates of the social and other costs of PHSMs, or brought together in a multicriteria decision making process[2]. Integrated epidemiological and economic modelling that considers health and cost impacts within a single framework has the potential to enhance planning and response strategies for future pandemics [3].

Infectious disease modelling can provide quantitative estimates regarding past or future response scenarios that have not been observed, based on available and projected data [4]. In a public health crisis, such as the recent COVID-19 pandemic, models can enhance our understanding of disease impacts on society, either unmitigated or in the presence of various interventions [5]. Dynamic transmission models allow for the risk of infection to be dependent on the prevalence of infectious individuals in the population, thereby capturing the indirect effects of infectious disease interventions and facilitating understanding of non-linear transmission effects [6–9]. Common types of dynamic models include compartmental models such as Susceptible-Infectious-Recovered (SIR) models, and agent-based models (ABMs).

Economic evaluation can be integrated into infectious disease modelling, providing an additional metric for comparison between intervention strategies. The perspective used in an economic evaluation can significantly influence the conclusions drawn regarding intervention impacts and policy recommendations and should therefore be selected with consideration of the specific context being modelled [10]. From a health system perspective, determining the most cost-effective intervention can facilitate resource allocation to mitigate morbidity and mortality resulting from a given disease. However, in a public health crisis such as the COVID-19 pandemic, impacts extend beyond the health system, necessitating the consideration of broader social and economic costs. Integrating economic evaluations, preferably extending beyond the health system to include broader societal impacts, into epidemiological models can help assess the proportionality of health responses and guide the selection of appropriate policy options [11].

Previous systematic reviews have summarised economic evaluations of pandemic disease intervention strategies [7, 11–20]. However, only two of these reviews have specifically examined the use of integrated epidemiological and economic models. These reviews focused on low- and middle-income settings [7], and on pandemic influenza [12]. A recent scoping review provided an evaluation of PHSMs against viral pandemics and had a similar focus but did not require the integration of dynamic transmission modelling. In Ref. [14] Therefore, we conducted a systematic review that builds upon the search strategy of Rasmussen and colleagues (2022) [14], narrowing the inclusion criteria by adding search terms for PHSMs and epidemiological models. The objective of our systematic review was to characterise publications that utilised integrated epidemiological and economic models to evaluate PHSMs against pathogens with pandemic potential.

## Methods

This systematic review was conducted in accordance with PRISMA guidelines.[21].

### Eligibility criteria and search strategy

We searched for studies that used a dynamic transmission model and incorporated an economic evaluation (reporting both cost and health impacts) of PHSMs (Table 1). Eligible studies modelled pathogens with pandemic potential (specifically Ebolavirus, Zika virus, influenza H1N1, influenza H5N1, MERS, SARS, or SARS-CoV-2 viruses).

**Table 1:** Inclusion and Exclusion Criteria

Inclusion criteria	Exclusion criteria
• Diseases of interest: infectious disease causing outbreaks or pandemics (including Ebolavirus, Zika virus, human influenza (H1N1 or H5N1), MERS, SARS-CoV-2, and SARS viruses) • Intervention: PHSMs directed at the disease of interest (including pharmaceutical and vaccine interventions if used in conjunction with PHSMs) • Dynamic transmission model (described in detail) with economic evaluation (reporting both cost and health impacts) of the PHSM(s) o For example, cost-effectiveness analysis, cost-utility analysis, or cost–benefit analysis	• Disease modelled is not a disease of interest • No dynamic simulation model was used • No details about how economic evaluation was used in the modelling • The economic evaluation only presents results for costs, without reporting health impacts • Published in a language other than English • Full-text unavailable • Commentaries, letters to the editor, editorials, unpublished grey literature, guidelines, reports, protocols, systematic reviews, literature reviews, and scoping reviews

Literature searches were conducted using Embase, PubMed, and Scopus from inception to the date of search, 7 July 2024. We narrowed the search strategy developed by Rasmussen et al.,[14] including search terms for PHSMs and dynamic transmission models (see Appendix 1 for search strategies).

All recovered citations were imported into Covidence, and duplicates were removed. Two reviewers (SR and SH) independently screened titles and abstracts of all citations for eligibility, followed by the full texts. During full-text review, articles were excluded hierarchically by assessing against exclusion criteria. The articles were excluded based on the first exclusion criteria of Table 1 that the record did not meet. Disagreements were resolved by a third reviewer (JS).

### Data analysis

We extracted data using a pre-designed template (Appendix 2) that included fields for: authors, year of publication, study location, study population, intervention administrative level, type of PHSM(s) modelled, type(s) of economic evaluation, optimal decision principle (the principle used by the authors to choose the most cost-effective intervention), dynamic model type and features, virus modelled, and funding source. Data were extracted by SR and reviewed by SH and JS. The data was analysed using frequency tables and narrative summaries.

## Results

**Fig. 1:**
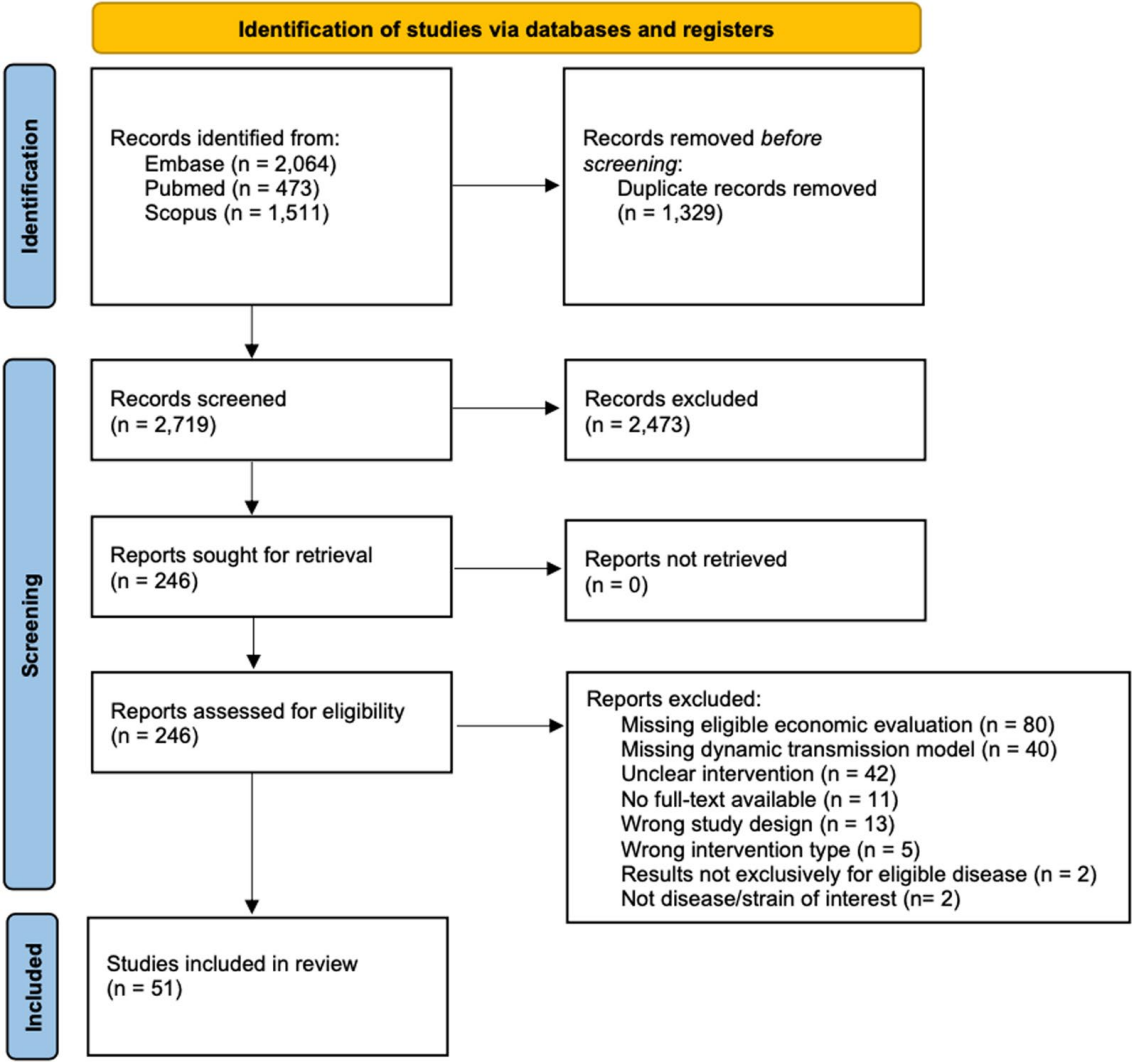
PRISMA Flow Diagram of Selected Studies

Our search identified 4,048 citations, which was reduced to 2,719 unique citations after duplicates were removed (Fig. 1). Of these, 51 citations met all eligibility criteria.

### Characteristics of the studies

The characteristics of the 51 eligible studies are summarised in Table 2. The studies were published between 2010 and 2024, with most (n = 43, 84%) published from 2020 onwards. Models were typically parameterised using available country-level demographic, cost, and disease transmission data. All continents were represented among eligible studies, with over one-third of the simulated epidemics occurring in North America (n = 19, 37%). Eligible analyses of MERS, Zika, and Influenza H5N1 were not identified. The majority of eligible studies modelled SARS-CoV-2 (n = 42, 82%), with approximately one-quarter of these (n = 10, 24%) modelling a specific SARS-CoV-2 variant, identified as beta [22], delta [23, 24], and omicron [25–31].

**Table 2:** Characteristics of included studies

Characteristic	Number of studies (n = 51)	%
Virus		
Ebolavirus	1 [32]	2
H1N1	7 [33–39]	14
SARS	1 [40]	2
SARS-CoV-2	42 [22–31, 41–72]	82
Continent		
Africa	2 [32, 57]	4
Asia	15 [22, 26, 27, 37, 38, 40, 49, 60, 61, 63, 64, 67, 68, 71, 72]	29
Europe	5 [39, 53, 58, 59, 62]	10
Global	4 [31, 43, 45, 70]	8
North America	19 [23, 24, 28–30, 33–35, 41, 42, 44, 48, 51, 52, 54–56, 66, 69]	37
Not specified	1 [65]	2
Oceania	5 [25, 36, 46, 47, 50]	10
Publication year		
< 2020	8 [33–40]	15
2020	4 [32, 42, 49, 56]	8
2021	18 [43, 46–48, 50–55, 57, 59–61, 63, 65, 66, 68]	35
2022	11 [22, 23, 25, 28, 41, 44, 45, 58, 62, 64, 67]	22
2023	5 [24, 26, 27, 69, 72]	10
2024^a^	5 [29–31, 70, 71]	10
Intervention ^b^		
Isolation/quarantine	13 [22, 26, 40, 42, 47, 48, 51, 57, 61, 63, 67, 68, 72]	25
Lockdowns	13 [22, 26, 41, 43, 45, 46, 50, 58–61, 67, 69]	25
Mask use	11 [22, 25, 41, 44, 46, 51, 62, 66, 68, 70, 71]	22
School closures	11 [25, 33–39, 46, 47, 69]	22
Social distancing	12 [25, 41, 43, 45–47, 51, 59, 61, 62, 67, 68]	24
Testing/screening	23 [23, 24, 26–31, 41, 42, 48, 49, 51, 52, 54–57, 62, 64, 65, 72]	45
Model type		
Compartmental model	37 [22, 24, 26, 32, 37–45, 47, 49–62, 65–72]	73
Agent-based model	12 [23, 25, 27–31, 34, 35, 46, 63, 64]	24
Other	2 [36, 48]	4
Code publicly available		
Yes	11[22, 24, 28, 29, 35, 43, 45, 52, 62, 65, 67]	22
No	40 [23, 25–27, 30–34, 36–42, 44, 46–51, 53–61, 63, 64, 66, 68–72]	78
Outcome metric		
ICER^^^	27 [28–31, 33, 37–42, 44, 51, 52, 54–57, 60, 61, 63, 64, 66, 69–72]	53
Net monetary benefit	8 [25–27, 46, 48, 49, 59, 68]	15
Cost per case averted	5 [23, 24, 34, 36, 62]	10
Cost per death averted	1[22]	2
Total cost	3 [35, 45, 67]	6
Other	7 [32, 43, 47, 50, 53, 58, 65]	14
Economic evaluation ^b^		
Cost-effectiveness	24 [22–24, 29, 31, 34, 36–38, 40, 42, 51–53, 55–57, 60–65, 72]	47
Cost-utility	17 [28, 30, 32, 33, 39, 41, 44, 48, 49, 51, 54, 60, 61, 66, 69–71]	33
Cost–benefit	17 [25–28, 34, 35, 43, 45, 47–50, 58, 59, 67, 68]	33
Perspective		
Health system	15[22, 29, 31, 40, 42, 47, 49, 54, 56, 57, 62, 64, 65, 70, 71]	29
Societal	5[33, 37, 52, 53, 60]	10
Societal and health system	30 [23–28, 30, 32, 34–36, 38, 39, 41, 44–46, 48, 50, 51, 55, 58, 59, 61, 63, 66–69, 72]	59
Not specified	1[43]	
		2

^a^ Until 7 July 2024 (date of search)

^b^ These categories are not mutually exclusive, as some evaluations employed multiple analysis types, or model packages of PHSMs

^^^ ICER: Incremental cost-effectiveness ratio

### Types of interventions

The PHSMs modelled in the included studies were isolation/quarantine, lockdowns, mask use, school closures, social distancing, and testing/screening policies (Tables 2 and 3). These intervention categories are not mutually exclusive, as many studies modelled packages of interventions (n = 28, 55%). Some studies also modelled PHSMs combined with pharmaceutical interventions [23–26, 33, 36, 59, 62]. A comparison of results between studies proved difficult due to substantial study heterogeneity, including differing outputs, timeframes, intervention specifications, and study populations.

**Table 3:** Summary of modelled interventions

Intervention category	Administrative level	Modelled in a package of interventions
Isolation/quarantine	National (n = 6) [40, 48, 61, 63, 68, 72] State (n = 1) [47] Sub-state^a^ (n = 6) [22, 26, 42, 51, 57, 67]	Yes (n = 13) [22, 26, 40, 42, 47, 48, 51, 57, 61, 63, 67, 68, 72] No (n = 0)
Lockdowns	National (n = 8) [41, 43, 45, 50, 58–61] State (n = 2)[46, 69] Sub-state^a^ (n = 3)[22, 26, 67]	Yes (n = 10) [22, 26, 41, 43, 45, 46, 50, 59, 61, 67, 69] No (n = 3) [50, 58, 60]
Mask use	National (n = 3) [41, 44, 68] State (n = 2) [25, 46] Sub-state^a^ (n = 6)[22, 51, 62, 66, 70, 71]	Yes (n = 10) [22, 25, 41, 44, 46, 51, 62, 66, 68, 70] No (n = 1) [71]
School closures	National (n = 2) [37, 38] State (n = 6) [25, 33, 34, 46, 47, 69] Sub-state^a^ (n = 3)[35, 36, 39]	Yes (n = 6) [25, 35, 36, 46, 47, 69] No (n = 5) [33, 34, 37–39]
Social distancing	National (n = 6)[41, 43, 45, 59, 61, 68] State (n = 3) [25, 46, 47] Sub-state^a^ (n = 3) [51, 62, 67]	Yes (n = 12) [25, 41, 43, 45–47, 51, 59, 61, 62, 67, 68] No (n = 0)
Testing/screening	National (n = 6) [27, 41, 48, 55, 65, 72] State (n = 1) [54] Sub-state^a^ (n = 16)[23, 24, 26, 28–31, 42, 49, 51, 52, 56, 57, 62, 64, 66]	Yes (n = 11) [24, 26, 29, 41, 42, 48, 51, 57, 62, 66, 72] No (n = 12) [23, 27, 28, 30, 31, 49, 52, 54–56, 64, 65]

^a^ The sub-state administrative level includes districts, counties, provinces, cities, and specific settings, such as hospitals and nursing homes

Testing/screening policies were the most frequently modelled intervention and were exclusively considered in SARS-CoV-2 models. Approximately half of these studies (n = 11, 48%) modelled testing/screening policies within a package of interventions (Table 3). This intervention was predominately modelled at a sub-jurisdictional administrative level in specific local settings (n = 16, 57%), rather than being implemented across an entire administrative region. These local contexts comprised, nursing homes[29, 30, 52], university campuses [24, 51, 52, 56, 66], homeless shelters [42], hospitals [62], schools [23, 31], sporting events [64], and workplaces [28].

School closures were typically modelled independently (n = 5, 45%) without incorporating other PHSMs. Notably, all H1N1 studies (n = 7) modelled school closure policies and were published between 2011 and 2016. Among SARS-CoV-2 studies, school closures were modelled in conjunction with other PHSMs [30, 35, 62]. This intervention was most frequently implemented at a state administrative level.

Isolation/quarantine, lockdowns, mask use, and social distancing measures were predominately modelled within a package of interventions in SARS-CoV-2 models. The only included SARS model, published in 2010, investigated quarantine strategies incorporating contact tracing measures at the national level in Hong Kong [40]. Isolation/quarantine and social distancing measures were exclusively modelled within packages of interventions. Face mask use was typically modelled in specific local settings (n = 6, 45%), such as university campuses [51, 66] and hospitals [62, 70, 71]. Studies that modelled lockdowns were primarily implemented at the national level (n = 8, 62%).

### Model designs

The characteristics of the epidemiological models are summarised in Table 2. Most included studies (n = 27, 53%) used an adaptation of the classic Susceptible-Exposed-Infectious-Recovered (SEIR)[22, 24, 26, 32, 39, 40, 42–45, 49, 50, 52, 54–57, 61, 62, 65–72]. These extended SEIR models can differentiate between various categories of infectiousness, with most included models (n = 37, 73%) explicitly accounting for asymptomatic infections [22–32, 35, 36, 39, 42, 44–46, 48, 49, 51, 54–59, 61, 62, 64–67, 69–72]. Additionally, most models (n = 40, 78%) incorporated a latent compartment to address the delay between infection and onset of infectiousness [22–25, 27–40, 42–46, 48, 52, 54–57, 59, 61–63, 65–68, 70–72]. Twelve studies used agent-based models (ABM), which more frequently accounted for vaccine introduction (n = 9, 75%) [23, 25, 27–31, 35, 64] and waning natural immunity from previous infection (n = 5, 42%) [25, 28, 29, 31, 35].

### Types of economic evaluation

Various methods for economic evaluation were reported in the eligible studies (Table 2), with some studies reporting multiple approaches. Cost-effectiveness analysis (e.g. cost per infection prevented) was the most frequently utilised, followed by cost-utility analysis (e.g. cost per quality-adjusted life year (QALY) gained), and cost–benefit analysis (e.g. monetised health gains using a net monetary benefit approach). Costing perspectives were also reported, with most studies (n = 30, 59%) considering both a health system and societal perspective. Considering both perspectives allowed the investigators to incorporate costs beyond the health sector. Lastly, when determining the most cost-effective intervention, most studies used an incremental cost-effectiveness ratio (ICER) (n = 27, 53%), followed by net monetary benefit (n = 8, 15%), and cost per case averted (n = 5, 10%).

## Discussion

Our review highlights the sparsity of integrated epidemiological and economic models used in the evaluation of PHSMs directed against infectious disease pathogens with pandemic potential. We identified 51 studies with disparate scopes. Most studies were published from 2020 onwards and modelled SARS-CoV-2 infection, indicating growth in the need for or interest in these interdisciplinary models during the pandemic era. However, given the importance of the subject matter and the volume of modelling conducted during the COVID-19 pandemic, relatively few published studies were found that addressed both health and economic impacts within a dynamic epidemiological model.

The COVID-19 pandemic has emphasised that pandemics generate both health and socioeconomic crises, requiring intersectoral collaboration for optimal policy implementation [3, 73]. Integrated epidemiological and economic models can be used as a tool to transparently assist in weighing socioeconomic trade-offs and support evidence-informed policy making [8, 74]. These interdisciplinary models can be used to appraise and compare multiple PHSMs. While aimed at reducing the risk and scale of transmission, the implementation of PHSMs frequently has unintended negative social and economic consequences [3]. Modelling multiple interventions, as done by approximately half of included studies, enables ranking of interventions against one another, or assessment of the effectiveness of the interventions as a ‘package’ against a comparator. In the context of a pandemic, wherein multiple PHSMs are implemented concurrently, this approach is likely to be more realistic [75]. The consideration of multiple interventions is therefore important for decision making in a pandemic, as society seeks to minimise health loss while avoiding unintended negative social and economic consequences.

Regarding health economic methods, most of the identified studies adopted a health system and societal costing perspective. A societal perspective estimates the broader costs to society, such as productivity loss [76]. The utilisation of both perspectives is valuable and complementary, as pandemics have societal and health system economic impacts. Moreover, PHSMs have broader macroeconomic implications than traditional healthcare treatments or pharmaceutical interventions [1].

A common limitation of the included studies was a lack of transparent reporting. Integrated epidemiological and economic models are inherently complex analyses; however, across the studies, the approach to reporting was highly variable. While reporting standards for this type of modelling remain lacking, documentation could have been enhanced by following existing guidelines for economic evaluation and dynamic transmission modelling [76, 77]. Nevertheless, the development of specific guidelines for integrated epidemiological and economic modelling is preferable. Moreover, only a few studies made their model code publicly available. Providing open access to code improves transparency and reproducibility of research, benefiting both scientific progress and clarity of model methodologies, and a rapid response in the event of a newly emerging pandemic.

Our review expands upon existing systematic reviews in the field of integrated epidemiological and economic modelling of pandemic interventions. In contrast to previous reviews, our search strategy imposed no geographical restrictions and encompassed multiple pathogens of pandemic potential while focusing on PHSMs. Additionally, we examined the administrative level of the modelled PHSMs and investigated whether packages of interventions were considered. Limitations of our review include that our search was confined to key databases that we deemed more likely to identify relevant studies. It is possible that other integrated epidemiological and economic models exist that have not been published. Further, non-English language studies were excluded by the search strategy, potentially omitting relevant studies.

The findings of our review can inform future work comparing and evaluating integrated epidemiological and economic model outputs. This may include a critical appraisal of specific PHSMs identified in this wider review, such as school closures or testing and screening policies, to enhance the understanding of their cost-effectiveness. An additional avenue of investigation may be to evaluate the timing of PHSM implementation and at which pandemic stage specific interventions are most cost-effective. Historical comparative SARS-CoV-2 data may be used to support this work. Furthermore, the development of an integrated epidemiological and economic modelling framework could facilitate the establishment of standardised methodologies at an international level to generate more comparable outputs and potentially expedite model construction when required.

## Conclusion

This systematic review demonstrated that a limited number of dynamic modelling studies of PHSMs have incorporated economic evaluation, and those identified varied in scope. In light of the COVID-19 pandemic, which necessitated rapid policy responses with substantial cost implications across multiple sectors of society, there is a need to expand the scope of this research going forward.

## Supplementary Information

Additional file 1.

## Data Availability

All data analysed during this study are included in this published article and its supplementary information files.

## Appendix 1

### Search strategies

#### Embase search details

Searched on 07/07/2024—2064 results.

1. Ebola hemorrhagic fever/
2. Zika fever/
3. “influenza a virus (h5n1)”/
4. Middle East respiratory syndrome/
5. severe acute respiratory syndrome/
6. “influenza a virus (h1n1)”/
7. (ebola or zika or H5N1 or mers or sars or H1N1).ti,ab.
8. (((pneumonia or covid* or coronavirus* or corona virus* or ncov* or 2019-ncov or sars*) adj5 Wuhan) or (SARS coronavirus* and Wuhan)).mp.
9. (2019-ncov or ncov19 or ncov-19 or 2019-novel CoV or sars-cov2 or sars-cov-2 or sarscov2 or sarscov-2 or Sars-coronavirus2 or Sars-coronavirus-2 or coronavirus-19 or covid19 or covid-19 or covid 2019 or "novel coronavirus" or "new coronavirus" or "nouveau coronavirus" or CoV or nCoV or covid or covid19 or covid-19 or "coronavirus 2" or ((novel or new or nouveau) adj1 (CoV or nCoV or covid or coronavirus* or "corona virus" or Pandemi*2)) or ((covid or covid19 or covid-19) and pandemic*2)).mp. [mp=title, abstract, heading word, drug trade name, original title, device manufacturer, drug manufacturer, device trade name, keyword, floating subheading word, candidate term word]
10. (COVID-19 or "severe acute respiratory syndrome 2" or 2019 pandemic or 2020 pandemic).mp. or SARS-like coronavirus*.ti,ab,ct.
11. or/1–10.
12. exp economic evaluation/
13. economic evaluation.ti,ab.
14. (cost* adj2 (effective* or utilit* or benefit* or minimi* or illness*)).ti,ab.
15. or/12–14.
16. (Simulation or model*).mp.
17. susceptible exposed infectious recovered model/ or model/ or mathematical model/ or susceptible infected recovered model/ or population model/ or theoretical model/or statistical model/ or stochastic model/ or epidemiological model/ or susceptible infected susceptible model/
18. or/17–18.
19. 11 and 15 and 18.
20. limit 16 to english language.

### Pubmed search details

#### Searched on 07/07/2024 – 473 results

(((((((("SARS Virus"[Mesh]) AND "Middle East Respiratory Syndrome Coronavirus"[Mesh]) AND "Ebolavirus"[Mesh]) AND "Zika Virus"[Mesh]) OR "Influenza A Virus, H1N1 Subtype"[Mesh]) OR "Influenza A Virus, H5N1 Subtype"[Mesh]) OR (SARS[Title/Abstract] OR MERS[Title/Abstract] OR Ebola[Title/Abstract] OR Zika[Title/Abstract] OR H1N1[Title/Abstract] OR H5N1[Title/Abstract])).

OR

(((“coronavirus”[MeSH Terms] OR “severe acute respiratory syndrome coronavirus 2”[Supplementary Concept] OR “coronavirus infections”[MeSH Terms] OR (“coronavirus*”[Title/Abstract] OR “coronovirus*”[Title/Abstract] OR “coronavirinae*”[Title/Abstract] OR “wuhan*”[Title/Abstract] OR “hubei*”[Title/Abstract] OR “huaian”[Title/Abstract] OR “2019 ncov”[Title/Abstract] OR “2019nCoV”[Title/Abstract] OR “nCoV2019”[Title/Abstract] OR “nCoV 2019”[Title/Abstract] OR “covid 19”[Title/Abstract] OR “COVID19”[Title/Abstract] OR “covid 19”[Title/Abstract] OR “HCoV 19”[Title/Abstract] OR “HCoV19”[Title/Abstract] OR “CoV”[Title/Abstract] OR “2019 novel*”[Title/Abstract] OR “ncov”[Title/Abstract] OR “n cov”[Title/Abstract] OR “SARS CoV 2”[Title/Abstract] OR “SARSCoV 2”[Title/Abstract] OR “SARSCoV2”[Title/Abstract] OR “SARS CoV2”[Title/Abstract] OR “SARSCov19”[Title/Abstract] OR “SARS Cov19”[Title/Abstract] OR “SARS Cov 19”[Title/Abstract] OR “novo”[Title/Abstract] OR “ncorona*”[Title/Abstract] OR “severe acute respiratory syndrome coronavirus 2”[Title/Abstract].

OR

“SARS2”[Title/Abstract] OR “2019 ncov”[Title/Abstract] OR “severe acute respiratory syndrome”[Title/Abstract] OR "SARS"[Title/Abstract])))))).

AND

(((“Cost–Benefit Analysis”[MeSH Terms] OR “economic evaluation”[Title/Abstract] OR “cost effectiv*”[Title/Abstract] OR “cost utility*”[Title/Abstract] OR “cost benefit*”[Title/Abstract] OR “cost minimi*”[Title/Abstract] OR “cost illness*”[Title/Abstract]))).

AND

(((“simulation*”[Title/Abstract] OR “model*”[MeSH Terms] OR “susceptible exposed infectious recovered model*”[Title/Abstract] OR “mathematical model*”[Title/Abstract] OR “susceptible infected recovered model*”[Title/Abstract] OR “population model*”[Title/Abstract] OR “theoretical model*”[Title/Abstract] OR “statistical model*”[Title/Abstract] OR “stochastic model*”[Title/Abstract] OR “epidemiological model*” [Title/Abstract] OR “susceptible infected susceptible model*”[Title/Abstract]))).

Filters: english

### Scopus search details

#### Adjusted search on 07/07/2024—1511 results

(TITLE-ABS-KEY (coronavirus OR "Middle East respiratory syndrome" OR "Severe acute respiratory syndrome" OR "Porcine epidemic diarrhea virus" OR "Feline infectious peritonitis virus" OR "Murine hepatitis virus" OR "Avian infectious bronchitis virus") OR TITLE-ABS-KEY (ebola OR zika OR h1n1 OR h5n1)).

AND

(TITLE-ABS-KEY (cost* PRE/2 effectiv*) OR TITLE-ABS-KEY (cost* PRE/2 utilit*) OR TITLE-ABS-KEY (cost* PRE/2 benefit*) OR TITLE-ABS-KEY (cost* PRE/2 illness*) OR TITLE-ABS-KEY (cost* PRE/2 analys*) OR TITLE-ABS-KEY (economic* PRE/2 evaluation*)).

AND

(TITLE-ABS-KEY (simulation OR model) OR TITLE-ABS-KEY (“Susceptible exposed infectious recovered model” OR “Susceptible infected recovered model” OR “Susceptible infected susceptible model”) OR TITLE-ABS-KEY (math* PRE/2 model*) OR TITLE-ABS-KEY (populat* PRE/2 model*) OR TITLE-ABS-KEY (theor* PRE/2 model*) OR TITLE-ABS-KEY (stat* PRE/2 model*) OR TITLE-ABS-KEY (stochastic* PRE/2 model*) OR TITLE-ABS-KEY (epidemiolog* PRE/2 model*)).

AND

(LIMIT-TO(LANGUAGE, "English")).

## Appendix 2

### Data extraction tool

The data extraction tool was used to assist in extracting relevant data from eligible studies. All reviewers used this tool.

1. Identifier and article information
  1.1 Initials of person extracting the data.
  1.2 Initials of second reviewer.
  1.3 Date of data extraction.
  1.4 Article identifier.
  1.5 Article title.
  1.6 First author’s last name.
  1.7 Year of publication.

2. Study design information
  2.1 Aim of the study.
  2.2 Study location.
  2.3 Study population.
  2.4 Administrative level of intervention.
  2.5 Intervention/s of interest.
  2.6 Were packages of interventions used?
  2.7 Comparator for intervention.

3. Economic evaluation
4. How was the optimal intervention chosen?
5. Was cost-effectiveness analysis used?
6. Was cost-utility analysis used?
7. Was cost–benefit analysis used?
8. Was cost-minimisation analysis used?
9. Was another type of economic evaluation used?
10. Time horizon.
11. Was discounting used?
12. Which perspective/s have been used when measuring costs?
13. Was a sensitivity analysis used?

4. Dynamic transmission model
5. Model type.
6. Infection.
7. Does the model have a latent compartment?
8. Does the model have an asymptomatic compartment?
9. Does the model allow for reinfections?
10. Does the model account for changes in the virus?
11. For SARS-CoV-2, is the variant being modelled specified?
12. Does the model account for vaccinated population/introduction of vaccines?
13. Does the model account for vaccine waning?
14. Does the model account for natural immunity?

5. Conclusions
  5.1 Key conclusions of study

6. Other
  6.1 Funding source.
  6.2 Is the model code provided?

## Abbreviations

ICER: Incremental cost-effectiveness ratio
NPI: Non-pharmaceutical intervention
PHSM: Public health and social measure
SEIR model: Susceptible-Exposed-Infectious-Recovered model
